# Entomopathogenic potential of bacteria associated with soil-borne nematodes and insect immune responses to their infection

**DOI:** 10.1371/journal.pone.0280675

**Published:** 2023-01-23

**Authors:** Ameni Loulou, Maristella Mastore, Sara Caramella, Aashaq Hussain Bhat, Maurizio Francesco Brivio, Ricardo A. R. Machado, Sadreddine Kallel

**Affiliations:** 1 Department of Plant Health and Environment, Laboratory of Bio-Aggressor and Integrated Protection in Agriculture, National Agronomic Institute of Tunisia, University of Carthage, Tunis, Tunisia; 2 Department of Theoretical and Applied Sciences, Laboratory of Comparative Immunology and Parasitology, University of Insubria, Varese, Italy; 3 Faculty of Sciences, Experimental Biology Research Group, Institute of Biology, University of Neuchâtel, Neuchâtel, Switzerland; University of Limpopo, SOUTH AFRICA

## Abstract

Soil-borne nematodes establish close associations with several bacterial species. Whether they confer benefits to their hosts has been investigated in only a few nematode-bacteria systems. Their ecological function, therefore, remains poorly understood. In this study, we isolated several bacterial species from rhabditid nematodes, molecularly identified them, evaluated their entomopathogenic potential on *Galleria mellonella* larvae, and measured immune responses of *G*. *mellonella* larvae to their infection. Bacteria were isolated from *Acrobeloides* sp., *A*. *bodenheimeri*, *Heterorhabditis bacteriophora*, *Oscheius tipulae*, and *Pristionchus maupasi* nematodes. They were identified as *Acinetobacter* sp., *Alcaligenes* sp., *Bacillus cereus*, *Enterobacter* sp., *Kaistia* sp., *Lysinibacillus fusiformis*, *Morganella morganii* subsp. *morganii*, *Klebsiella quasipneumoniae* subsp. *quasipneumoniae*, and *Pseudomonas aeruginosa*. All bacterial strains were found to be highly entomopathogenic as they killed at least 53.33% *G*. *mellonella* larvae within 72h post-infection, at a dose of 10^6^ CFU/larvae. Among them, *Lysinibacillus fusiformis*, *Enterobacter* sp., *Acinetobacter* sp., and *K*. *quasipneumoniae* subsp. *quasipneumoniae* were the most entomopathogenic bacteria. Insects strongly responded to bacterial infection. However, their responses were apparently little effective to counteract bacterial infection. Our study, therefore, shows that bacteria associated with soil-borne nematodes have entomopathogenic capacities. From an applied perspective, our study motivates more research to determine the potential of these bacterial strains as biocontrol agents in environmentally friendly and sustainable agriculture.

## Introduction

Over the past decades, entomopathogenic bacteria (EPBs) and their toxins have been successfully commercialized as microbial insecticides and used in biological control programs as eco-friendly and sustainable alternatives to synthetic pesticides [1,2]. Several entomopathogenic bacteria species from different families such as *Bacillaceae*, *Pseudomonadaceae*, *Enterobacteriaceae*, *Streptococcaceae*, *Micrococcaceae*, *Paenibacillaceae*, *Neisseriaceae*, *Rickettsiaceae* and *Coxiellaceae* are commonly used [3–5]. In this context, Bacillus thuringiensis, *Lysinibacillus sphaericus*, Paenibacillus popilliae, *Yersinia entomophaga*, *Chromobacterium subtsugae*, and *Burkholderia rinojensis* have received the most attention as microbial control agents [6–9]. Furthermore, species of *Xenorhabdus* and *Photorhabdus* stand out for their entomopathogenic potential [10,11]. They are mutualistically associated with entomopathogenic nematodes (EPNs) of the genera *Steinernema* and *Heterorhabditis*, respectively [12–14]. Both genera bacteria colonize the intestines of the dauer larvae of EPNs [15–17]. Their insecticidal potential is mostly based on the secretion of toxic compounds into the hemocoel that suppress the insect immune responses, causing host death [18,19]. Also some species of *Serratia* show a highly lethal effect on insects [20–22]. For example, *Serratia* sp. isolated from *Caenorhabditis briggsae* (Rhabditida: Rhabditidae) is extremely pathogenic to *Galleria mellonella* [23]. In addition, bacteria of the genus *Serratia* is frequently found in association with other nematode species such as *Oscheius carolinensis*, *O*. *chongmingensis*, and *O*. *rugaoensis*, and have been isolated from the gut of the infective juvenile (IJ) and from the cuticle of these nematodes [24–28]. These bacteria have also been shown to be entomopathogenic [26]. *Oscheius* nematodes associate also with other entomopathogenic bacteria such as *B*. *cereus*, *Ochrobactrum tritici*, *Flavobacterium* sp., *Providencia vermicola*, and *S*. *marcescens* [29–32]. Clearly, nematodes may be a suitable source of bacteria with entomopathogenic potential. The efficacy of EPBs can be assessed by studying the immunological responses of host insects which are composed by two closely interconnected reactions: cellular and humoral defenses [33,34]. Insect hemocytes mediate cellular immune responses, including processes such as, phagocytosis, nodulation, encapsulation, and production of antimicrobial compounds [33,35–37]. Immunocompetent cells circulate freely in the insect hemolymph and can also be found in the digestive tract and fat bodies [36,38,39]. Different specialized cells prohemocytes, plasmatocytes, granulocytes, spherulocytes and oenotocytes are the most common types of hemocytes [40,41]. Their number in the hemolymph can vary during bacterial infection. They can for instance increase due to migration from different insect organs and tissues to the hemolymph. They can also decrease due to phagocytosis, nodulation and/or encapsulation processes [36]. Different type of hemocytes act in concert in the process of nodulation, which eliminates a large number of pathogens by forming layers of cells around them. This response is preceded by the activation of phenoloxidases, which in turn, leads to the melanization of nodules [33,40]. In the encapsulation process, granular cells recognize large foreign bodies in the hemolymph, and promote plasmatocyte binding. When capsule formation begins, the number of hemocytes circulating in the hemolymph decreases and increases after capsule formation [42]. Besides, pathogens could be destroyed by enzymes released by hemocytes through degranulation, in the process of cytotoxicity [36,43]. Stimulation of insect humoral responses after infection leads to the synthesis of a pool of antimicrobial peptides (AMPs) such as moricin-like peptides, cecropins, gloverin, Gm proline-rich peptides, Gm anionic peptides, galiomycin, gallerimycin, inducible serine protease inhibitors, defensins, cobatoxins, and heliocin-like peptides [44–47]. Certain immune-relevant proteins and peptides, including lysozyme, apolipophorins, and anionic peptides, are normally present in the hemolymph of healthy larvae and their concentration could be altered after pathogen infection [45,48]. Lysozyme acts on the peptidoglycan layer present in fungi and bacteria [36,49,50]. These enzymes participate in the cellular and humoral immune responses and affect host–parasite interactions [51,52]. During a microbial infection, prophenoloxidase cascade (proPO system), released from various hemocytes, is activated by serine proteases [53]. In its activated form, PO converts tyrosine to dihydroxyphenylalanine (DOPA) and oxidizes phenolic substances to quinones and melanin, in a process that often induces the darkening of infected larvae [54–56]. Moreover, proPO system activation can lead to synthesis of antimicrobial peptides and activation of some cellular immune responses [57,58].

In this study, we isolated several bacterial strains from different soil-borne nematode species, tested their entomopathogenic potential, and evaluated the immune responses of insects to their infection. Our study highlights a promising potential of several bacterial strains to be used as bioinsecticides for the biological control of agricultural pests.

## Materials and methods

### Bacteria isolation

Bacterial strains were obtained from ten soil-borne nematode strains: *Oscheius tipulae* OC2 and TC2, *Acrobeloides* sp.TC9, TG13A, and HW0, *Acrobeloides bodenheimeri* TC7 and KG18, *Pristionchus maupasi* IN18 and IN7, *Heterorhabditis bacteriophora* EN01, and from an unidentified soil-borne nematode, IN1 (Table 1). The nematode isolation procedure is described in Loulou *et al*., (2022) [59]. To isolate the bacteria from all but IN1 nematodes, approximately 200 specimens of each nematode strain were washed several times with sterile phosphate-buffered saline (PBS, 1mM KH_2_PO_4_, 1mM K_2_HPO_4_, 5M NaCl, pH 7.2). Then, the nematodes were incubated in a 1% v/v sodium hypochlorite/PBS solution for 5 min under gentle orbital agitation. After this incubation period, the nematodes were recovered by decanting, washed with sterile PBS, and incubated again in a 1% v/v sodium hypochlorite/PBS solution. After this incubation period, the nematodes were again recovered by decanting, washed several times with PBS, and sonicated in sterile PBS at 4°C for 30 sec and at 100W burst using an ultrasonic processor (Labsonic-L, B-Braun Biotech Inc, Allentown, PA, USA). The nematodes body fragments were pelleted by centrifugation at 650g (10 min, 4°C). Then, 100 μL of supernatants were cultured in LB medium overnight at 30°C under constant shaking (180 rpm). An aliquot of the resulting cultures was plated on LB solid agar and incubated at 30°C for 24–48 h. Single colonies were sub-cultured and used for further experiments. Bacteria associated with IN1 nematodes were isolated by plating drops of hemolymph from *Galleria mellonella* larvae on LB agar plates. For this, larvae were dipped first in 70% ethanol, and then punctured with a sterile needle in the ventral part. The released hemolymph was cultured in LB broth and incubated overnight at 30°C for 24h under constant shaking (180 rpm). An aliquot of the resulting cultures was plated on LB agar and incubated at 30°C for 24–48 h. Single colonies were sub-cultured and used for further experiments. Bacterial stocks were stored at -80°C in LB supplemented with 20% (v /v) glycerol. The obtained bacterial strains were named using the initial letters of their genus and species/subspecies as a prefix and then using the strain name of their nematode host (Table 1). Hence, the strain designations are Lf_OC2, K_TC2, A_TC9, E_TC7, Kqq_KG18, Bc_IN18, Bc_IN7, A_IN1, Bc_TG3A, Pa_HWO, and Mmm_EN01 (Table 1).

**Table 1:** Characteristics of the bacterial strains isolated in this study.

Code	Bacterial ID	Associated nematode	Place of nematode	Insect host
Lf_OC2	*Lysinibacillus fusiformis*	*Oscheius tipulae* OC2	Ouzra	*C*. *capitata* pupae
K_TC2	*Kaistia* sp.	*Oscheius tipulae* TC2	Takilsa	*C*. *capitata* pupae
A_TC9	*Alcaligenes* sp.	*Acrobeloides tricornis* TC9	Takilsa	*C*. *capitata* pupae
E_TC7	*Enterobacter* sp.	*Acrobeloides bodenheimeri* TC7	Takilsa	*C*. *capitata* pupae
Kqq_KG18	*Klebsiella quasipneumoniae*s ubsp. *quasipneumoniae*	*Acrobeloides bodenheimeri* KG18	Kobba	*G*. *mellonella* larvae
Bc_IN18	*Bacillus cereus*	*Pristionchus maupasi* IN18	INAT-Tunis	*G*. *mellonella* larvae
Bc_IN7	*Bacillus cereus*	*Pristionchus maupasi* IN7	INAT-Tunis	*G*. *mellonella* larvae
A_IN1	*Acinetobacter* sp.	Unidentified sp. IN1	INAT-Tunis	*G*. *mellonella* larvae
Bc_TG3A	*Bacillus cereus*	*Acrobeloide s*sp.TG3A	Takilsa	*G*. *mellonella* larvae
Pa_HWO	*Pseudomonas aeruginosa*	*Acrobeloides tricornis* HWO	Chott-Meriem	Locust egg
Mmm_EN01	*Morganella morganii* subsp. *morganii*	*Heterorhabditis bactriophora* EN01	E-nema Germany	*G*. *mellonella* larvae

### Bacteria identification

Bacterial strains were identified based on 16S rRNA gene sequences and/or whole genome sequences using the EzBioCloud’s identification service and The Type (Strain) Genome Server (TYGS), two free bioinformatics platforms available under https://www.ezbiocloud.net and https://tygs.dsmz.de, respectively [60,61]. Whole genome sequences were obtained as described [62]. Briefly, genomic DNA was extracted and purified using the GenElute Bacterial Genomic DNA Kit (Sigma–Aldrich, Switzerland) following the manufacturer’s instructions. The resulting DNA was used for library preparation using the TruSeq DNA PCR–Free LT Library Prep (FC–121–3003) kit. Indexed libraries were then pooled at equimolar concentrations and sequenced (2 × 150 bp) on an Illumina HiSeq 3000 instrument. Genomes were assembled using the Bactopia pipeline [63]. To this end, the raw Illumina reads were quality trimmed using Trimmomatic 0.39 [64]. The resulting reads were assembled with SPAdes 3.14.1 (*k*–mer sizes of 31, 51, 71, 91, and 111 bp) [65]. Scaffolds with a mean read–depth smaller than 20% of the median read–depth of the longer scaffolds (≥ 5,000 bp) as well as scaffolds that were shorter than 200 bp were removed. The final assemblies were polished using Pilon 1.22 [66].

To obtain 16S rRNA gene sequences, the 16S rRNA gene was amplified by polymerase chain reaction (PCR) using the following universal primers: 27F (5’-AGAGTTTGATCMTGGCTCAG-3’) and 1525R (5’-AAGGAGGTGWTCCARCC-3’) and the following cycling conditions: 1 cycle at 94°C for 10 min followed by 40 cycles at 94°C for 60 s, 55°C for 60 s, 72°C for 60 s and a final extension at 72°C for 5 min. PCR products were separated by electrophoresis in a1% TAE-agarose gel stained with GelRed nucleic acid gelstain (Biotium), gel-purified (QIAquick gel purification kit Qiagen) and sequenced by Sanger sequencing (Microsynth). Obtained16S rRNA raw sequences were manually curated using Bioedit 7.2.5 [67].

### Bacterial pathogenicity against *G*. *mellonella* larvae

To evaluate the entomopathogenic potential of the isolated bacteria, *G*. *mellonella* larvae were injected with the different bacterial strains at different bacterial concentrations. To this end, overnight cultures were centrifuged at 1500g for 20 min. Bacterial pellets were then washed several times with sterile PBS. For larvae infection, three bacterial concentrations, 10^2^, 10^4^, and 10^6^ CFU/larva, obtained by serial dilutions, were used. Five μl of bacterial solution were injected in the abdominal spiracle of the larvae using a sterile gas-tight syringe with a 30 gauge hypodermic needle (Hamilton Co, Reno, NE, USA). Ten surface-sterilized last instar larvae per bacterial strain and concentration were injected. Controls consisted of larvae injected with PBS buffer. The larvae were placed in sterile plastic boxes and kept in the dark at 25°C. Insect mortality was determined at 12, 24, 48 and 72h post-injection. All assays were carried out in triplicate.

### Immune response of *G*. *mellonella* larvae to bacterial infection

#### Hemolymph collection

To collect hemolymph for hemocyte number determination, and to measure phenoloxidase and lysozyme activity, last-instar *G*. *mellonella* larvae were injected with the different bacterial strains at a concentration of 10^2^ CFU/larva as described above. Then, the larvae were incubated in sterile plastic boxes and kept in the dark at 25°C. Larvae were collected 4, 24, and 48h after injections, anesthetized on ice, surface-sterilized with 70% ethanol, and punctured with a sterile needle in the ventral region. Released hemolymph was collected in siliconized Eppendorf microcentrifuge tubes, and centrifuged at 250g for 5 min at 4°C to separate humoral fraction from hemocytes. In all samples few crystals of phenylthiourea (PTU) were added to prevent melanization, except those samples used for phenoloxidase activity assays. Samples of cell-free hemolymph and hemocytes were used for phenoloxidase and lysozyme activity, and total hemocyte counts. All assays were carried out at 4, 24, and 48h post-infection with three replicates. Controls included hemolymph from non-injected and PBS-injected larvae [68].

#### Total hemocyte counts (THC)

To determine the total number of hemocytes present in hemolymph samples, 10 μL of hemolymph were disposed on a Neubauer hemocytometer, counting the hemocytes a Corning Cell counter (Corning Inc., NY, USA) at a magnification of 5X. The data obtained were processed by CytoSmart^®^ [68].

#### Phenoloxidase activity

Relative phenoloxidase activity was determined by measuring the absorbance spectrophotometrically (Jasco V-560 spectrophotometer, Easton, MD, USA) at 490 nm, every 5 min, for 30 min, using L-Dopa (L-3,4-Dihydroxyphenylalanine) as substrate. Phenoloxidase activity was recorded as the formation of dopachrome from L-Dopa substrate and expressed in absorbance units per μl of hemolymph (**Δ**Abs_490_/30min/10μl). All the assays were carried out with 5μl of hemolymph in 995μl of L-Dopa buffer (8 mM L-Dopa in 10 mM Tris-HCL pH 7.2) [69].

#### Lysozyme activity

Hemolymph lysozyme activity was evaluated by their lytic action on *Micrococcus luteus* [70]. Assays were carried out by turbidimetry changes in samples measuring the decrease of absorbance induced by lysozyme cell lysis. Absorbance was recorded by a Bio Rad iMark™ Microplate Absorbance reader, at 450 nm, for 10 min, in hemolymph collected from larvae at 4, 24 and 48h after bacterial injections. One unit of lysozyme activity is defined as the change of 0.001 units/min of a suspension of *M*. *luteus*. For each sample, three replicates were carried out, and *M*. *luteus* suspension, hemolymph from PBS-injected and non-injected larvae were used as control [69].

### Chemicals and instruments

All reagents were supplied by Sigma Chemicals (St. Louis, MO, USA), Merck Millipore Ltd. (Tullagreen, Cork, Ireland). Instruments were provided by Bio-Rad Laboratories (Detroit, MI, USA) and Euroclone S.p.A. (Milan, Italy, EU). Centrifugations were carried out with a SIGMA 1–14 (SciQuip Ltd., Newtown, Wem, Shropshire, UK) microcentrifuge and Eppendorf 5804 (Eppendorf, AG, Hamburg, Germany) centrifuge. Spectrophotometric analysis was performed with a Jasco V-560 spectrophotometer (Easton, MD, USA). All materials, buffers, and solutions were autoclaved or filtered with 0.22 μm Minisart filters (Sartorius, Goettingen, Germany).

### Statistical analysis

Differences in the bacterial pathogenicity against *G*. *mellonella* larvae and in insect immunological responses to the different bacterial strains were assessed by one-way ANOVA. Normality and equality of variance were verified using Shapiro–Wilk and Levene’s tests at P = 0.05. In some cases, square transformation was performed. Duncan post-hoc test was used for multiple comparisons. Dunnett tests were carried out to compare treatment data to control data with a probability of 0.05. Kruskall and Wallis tests were carried out to compare data across all treatments with P = 0.05. All statistical analyses were conducted using SPSS software 16.0^®^ [71,72].

## Results

### Bacteria identification

Based on the analysis of 16S rRNA gene sequences and/or whole genome sequences the bacteria isolated from the different nematode isolates were identified as *Lysinibacillus fusiformis*, *Kaistia* sp., *Alcaligenes* sp., *Enterobacter* sp., *Klebsiella quasipneumoniae* subsp. *quasipneumoniae*, *Bacillus cereus* (three different strains), *Acinetobacter* sp., *Pseudomonas aeruginosa*, and *Morganella morganii* subsp. *morganii* (Table 1, S1 Fig). The bacterial strains identified as *Kaistia* sp., *Alcaligenes* sp., *Enterobacter* sp., and *Acinetobacter* sp., represent new species, which will be formally described elsewhere (Table 1).

### Entomopathogenic potential

**Fig 1:**
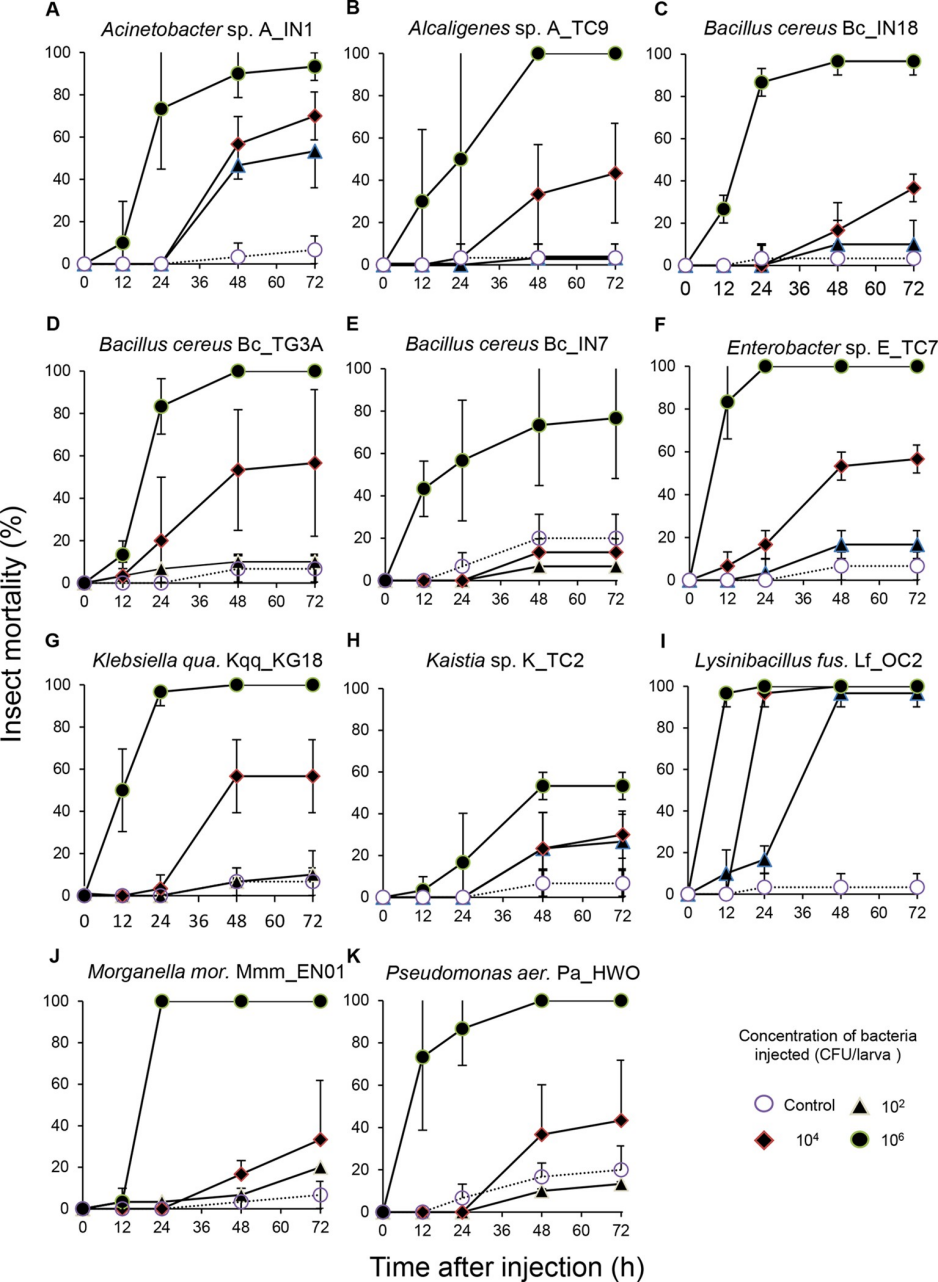
Entomopathogenic potential of different bacterial strains against G. mellonella larvae. Insects were injected with three bacterial concentrations 10^2^, 10^4^, or 10^6^ CFU/larva of the following bacterial strains. A) *Acinetobacter* sp. A_IN1. B) *Alcaligenes* sp. A_TC9. C) *Bacillus*. *cereus* Bc_IN18. D) *Bacillus cereus* Bc_TG3A. E) *Bacillus cereus* Bc_IN7. F) *Enterobacter* sp. E_TC7. G) *Klebsiella quasipneumoniae* subsp. *quasipneumoniae* Kqq_KG18. H) *Kaistia* sp. K_TC2. I) *Lysinibacillus fusiformis* Lf_OC2. J) *Morganella morganii* subsp. *morganii*. Mmm_EN01. K) *Pseudomonas aeruginosa* Pa_HWO. Bars correspond to confidence intervals (P = 0.05).

When injected into the hemocoel of *G*. *mellonella* larvae, all bacteria effectively killed these insects in a bacterial strains, time, and concentration dependent manner (Table 2). When the bacteria were injected at a concentration of 10^6^ CFU/insect, most of them killed more than 80% of the insects within 48h. Some bacteria were more pathogenic and killed more than 80% of the insects even within 24h post injection. This was the case of: *B*. *cereus* Bc_IN18, *B*. *cereus* Bc_TG3A, *Enterobacter* sp. E_TC7, *K*. *quasipneumoniae* Kqq_KG18, *L*. *fusiformis* Lf_OC2, and *M*. *morganii* Mmm_EN01 (Fig 1, Table 2). Some other bacteria, such as *B*. *cereus* Bc_IN7 and *Kaistia* sp. K_TC2, were much less pathogenic and killed no more than 50–80% of the insects within 72h at the same bacterial concentration. When the bacteria were injected at concentrations of 10^4^ CFU/larva, lower mortalities were caused by most of the bacterial strains (Fig 1). Notably, *L*. *fusiformis* Lf_OC2 still killed 90% of the insects within 24h, while *Acinetobacter* sp. A_IN1, *Enterobacter* sp. E_TC7, *B*. *cereus* Bc_TG3, and *K*. *quasipneumoniae* Kqq_KG18 killed between 50-70% of the insects within 72h (Fig 1, Table 2). When bacteria were injected at a concentration of 10^2^ CFU/larva, only *L*. *fusiformis* Lf_OC2 killed up to 90% of the insects within 72h. At the same concentration, *Acinetobacter* sp. A_IN1 killed about 50% of the insects, while the other bacterial strains were ineffective and killed less than 30% of the insect within 72h post-treatment (Fig 1, Table 2).

**Table 2:** Virulence of bacteria isolates against *Galleria mellonella* larvae at 72h post-treatment and time (h) required to obtain 50% (TL50) and 90% (TL90).

	Dose CFU/larva		
	10^2^	10^4^	10^6^
*Lysinibacillus fusiformis*	%**M** 96.66 a*	100 a*	100 a*
	**TL50** 33.2	17.5	5.8
	**TL90** 44.5	22	10
*Kaistia* sp.	%**M** 26.66 c*	30.00 de	53.33 c*
	**TL50 -**	**-**	45
	**TL90 -**	**-**	**-**
*Alcaligenes* sp.	%**M** 3.33 e	43.33 cd*	100 a*
	**TL50 -**	**-**	24
	**TL90 -**	**-**	42,5
*Enterobacter* sp.	%**M** 16.66 cde	56.66bc*	100 a*
	**TL50 -**	45	6
	**TL90 -**	**-**	15
*Klebsiella quasipneumoniae* subsp. *quasipneumoniae*	%**M** 10.00 de	56.66 bc*	100 a*
	**TL50 -**	44	12
	**TL90 -**	**-**	20
*Bacillus cereus* Bc_IN18	%**M** 10.00 de	36.66 cd*	96.66 a*
	**TL50 -**	**-**	16,2
	**TL90 -**	**-**	27
*Bacillus cereus* Bc_TG13 A	%**M** 10.00 de	56.66 bc*	100 a*
	**TL50 -**	46.8	17.2
	**TL90 -**	**-**	17
*Acinetobacter* sp.	%**M** 53.33 b*	70.00 b*	93.33 a*
	**TL50** 52,4	44,8	18,2
	**TL90 -**	**-**	48
*Bacillus cereus* Bc_IN7	%**M** 6.66 de	13.33 ef*	76.66 b*
	**TL50-**	**-**	15
	**TL90 -**	**-**	**-**
*Pseudomonas aeruginosa*	%**M** 13.33 cde	43.33 cd*	100 a*
	**TL50 -**	**-**	7.2
	**TL90 -**	**-**	30
*Morganella morganii* subsp. *morganii*	%**M** 20.00 cd	33.33 de*	100 a*
	**TL50 -**	**-**	17
	**TL90 -**	**-**	21
**Control**	%**M** 8.18de	8.18 f	8.18 d
	**TL50 -**	**-**	**-**
	**TL90 -**	**-**	**-**

The means by bacteria followed by the same letters (a, b) are not significantly different according to the test of Duncan and Kruskall and Wallis with P < 0.05.The means by bacteria followed by (*) are not significantly different according to the test of Dunnett(P = 0.050).

### The immune response of larvae to infection

#### Total hemocytes counts (THC)

**Fig 2:**
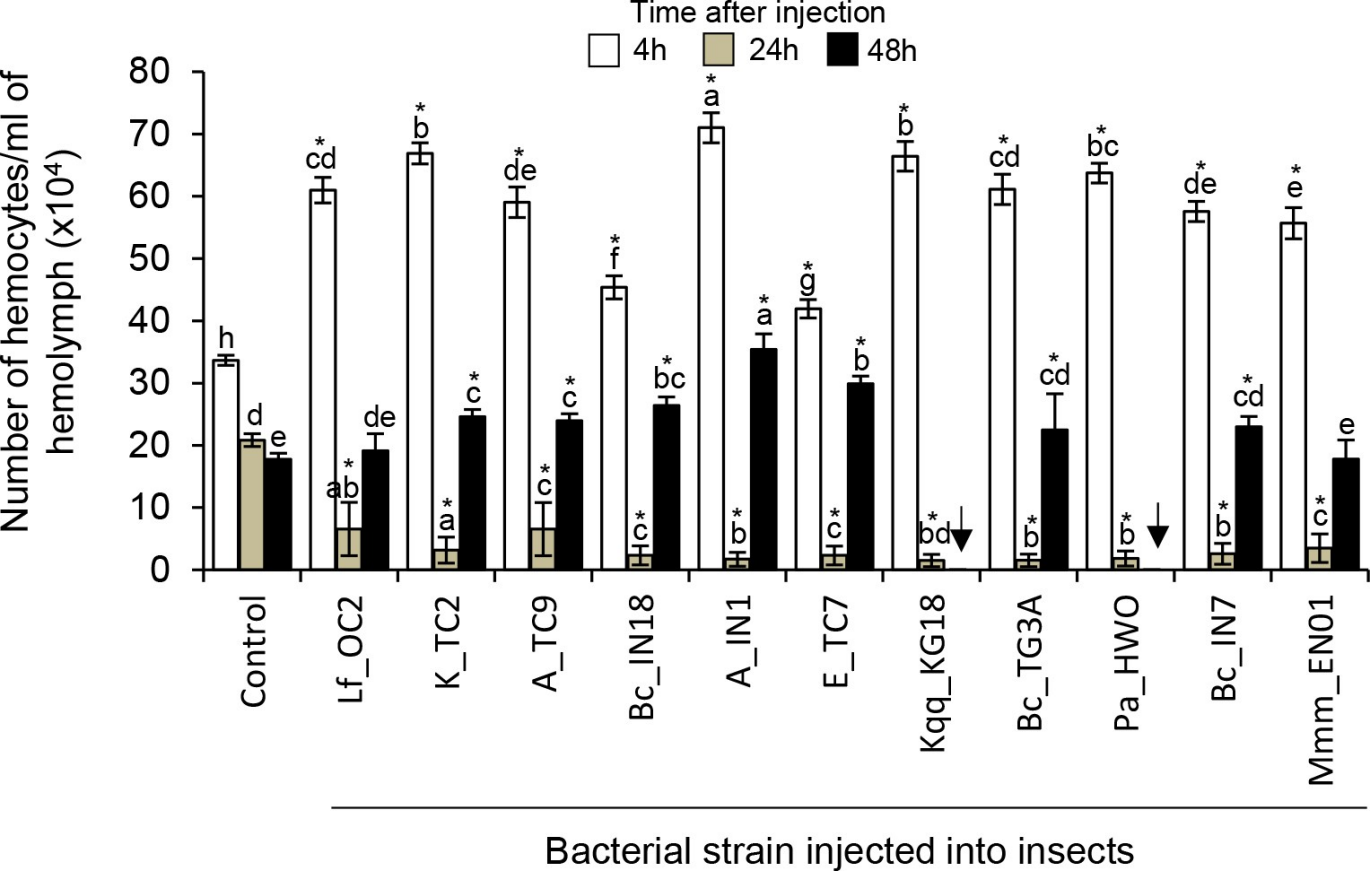
Total Hemocyte Counts (THC) in bacteria-infected insects. Mean hemocyte number per ml of hemolymph of *G*. *mellonella* larvae injected with different bacteria or with buffer as controls at 4h, 24h and 48h post-treatment. Bars correspond to confidence intervals (P = <0.05). Different letters indicate significant differences between the number of hemocytes across all treatments within each time point (Duncan test, Kruskall, and Wallis test, P<0.05). Asterisks indicate significant differences in the number of hemocytes of control larvae and bacteria-injected larvae within each time point (Dunnet test, P<0.05). Arrows indicate that the insects died.

The number of hemocytes in the hemolymph of bacteria-injected larvae increased 4h post-infection (Fig 2). In some cases, however, the number of hemocytes was only slightly higher in bacteria-injected larvae compared to controls, i.e. with insects injected with *B*. *cereus* Bc_IN18 and *Enterobacter* sp. E_TC7. The highest number of hemocytes was found in the hemolymph of insects injected with *Acinetobacter* sp. A_IN1. The number of hemocytes decreased sharply 24h post-infection, and only minor differences in the number of hemocytes were observed. 48h post-infection, the number of hemocytes increased again and was slightly higher in insects injected with most of the bacterial strains, apart from those injected with *L*. *fusiformis* Lf_OC2 and *M*. *morganii* subsp. *morganii* Mmm_EN01.

#### Lysozyme activity

**Fig 3:**
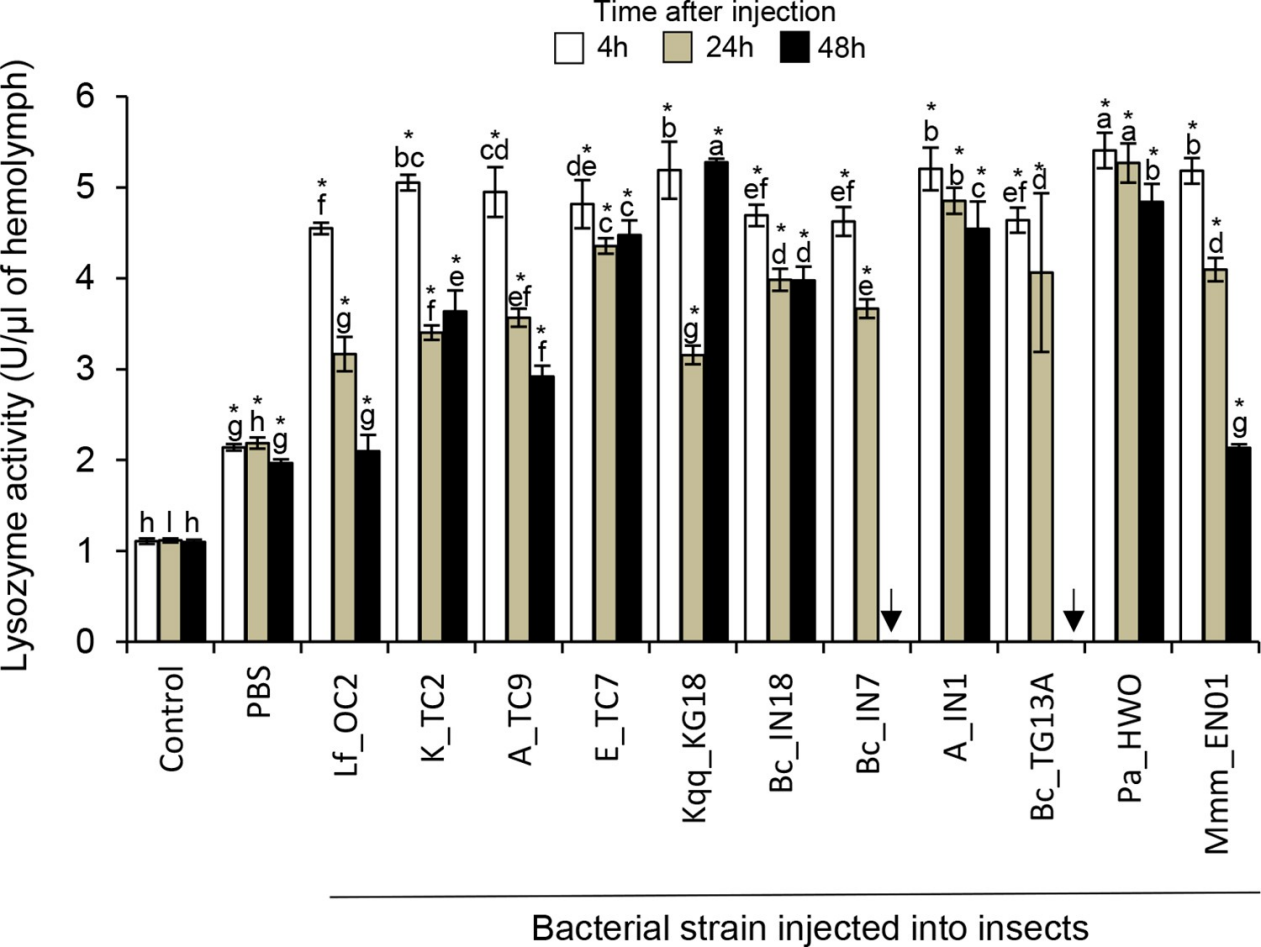
Lysozyme activity in the hemolymph of bacteria-infected insects. Mean lysozyme activity in the hemolymph of *G*. *mellonella* larvae injected with different bacteria or with buffer as controls at 4h, 24h and 48h post-treatment. Bars show confidence intervals (P = 0.05). Different letters indicate significant differences in lysozyme activity across all treatments within each time point (Duncan test, Kruskall and Wallis test, P<0.05). Asterisks indicate significant differences in lysozyme activity between control larvae and bacteria-injected larvae within each time point (Dunnet test, P<0.05). Arrows indicate that the insects died.

Lysozyme activity in the hemolymph of bacteria-injected larvae increased 4h post-infection (Fig 3). There were no major differences in enzymatic activity in the hemolymph of insects injected with the different bacteria 4h post-infection. The highest enzymatic activity was observed in the hemolymph of insects injected with *L*. *fusiformis* Lf_OC2, the lowest activity was observed in the hemolymph of insects injected with *P*. *aeruginosa* Pa_HWO. 24h post-infection, lysozyme activity decreased in most cases, but remained at similar levels as those observed 4h post-infection in the hemolymph of insects injected with *Enterobacter* sp. E_TC7, *Acinetobacter* sp. A_IN1, and *P*. *aeruginosa* Pa_HWO. The highest enzymatic activity was observed in the hemolymph of insects injected with *Acinetobacter* sp. A_IN1 and *P*. *aeruginosa* Pa_HWO, the lowest was observed in the hemolymph of insects injected with *L*. *fusiformis* Lf_OC2. 48h post-infection, the enzymatic activity remained at similar levels as those observed 24h post-infection, although it decreased in the hemolymph of insects injected with *L*. *fusiformis* Lf_OC2 and *M*. *morganii* subsp. *morganii* Mmm_EN01, and increased in the hemolymph of insects injected with *K*. *quasipneumoniae* subsp. *quasipneumoniae* Kqq_KG18. The highest enzymatic activity was observed in the hemolymph of insects injected with *K*. *quasipneumoniae* subsp. *quasipneumoniae* Kqq_KG18, the lowest was observed in the hemolymph of insects injected with *L*. *fusiformis* Lf_OC2 and *M*. *morganii* subsp. *morganii* Mmm_EN01 (Fig 3).

#### Phenoloxidase activity

**Fig 4:**
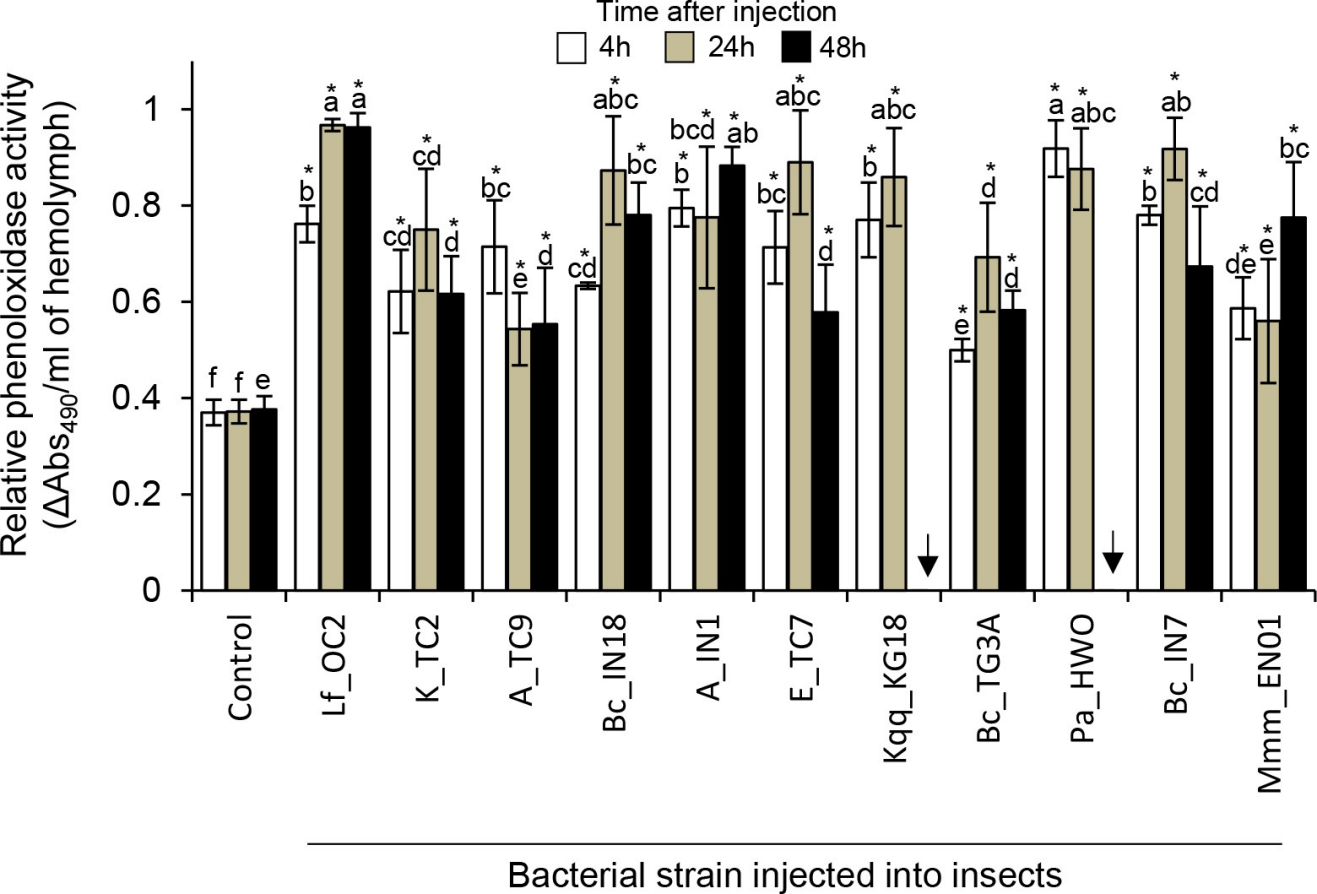
Relative phenoloxidase activity in the hemolymph of bacteria-infected insects. Mean relative phenoloxidase activity per ml of hemolymph of *G*. *mellonella* larvae injected with different bacteria or with buffer controls at 4h, 24h, and 48h post-treatment. Absorbance measurements were taken 30 min after hemolymph incubation. Bars correspond to the confidence intervals at P = 0.05. Different letters indicate significant differences in phenoloxidase activity across all treatments within each time point (Duncan test, Kruskall and Wallis test, P<0.05). Asterisks indicate significant differences in phenoloxidase activity between control larvae and bacteria-injected larvae within each time point (Dunnet test, P<0.05). Arrows indicate that the insects died.

Phenoloxidase activity in the hemolymph of bacteria-injected larvae increased 4h post-infection and remained higher in the hemolymph of bacteria-injected larvae than in the hemolymph of control, non-injected insects for 48h post-infection (Fig 4). There were no major differences in enzymatic activity in the hemolymph of insects injected with the different bacteria between 4-48h post-infection. The highest enzymatic activity was observed in the hemolymph of insects injected with *L*. *fusiformis* Lf_OC2 (Fig 4).

### Relationships between insect immune responses and bacteria pathogenesis

**Fig 5:**
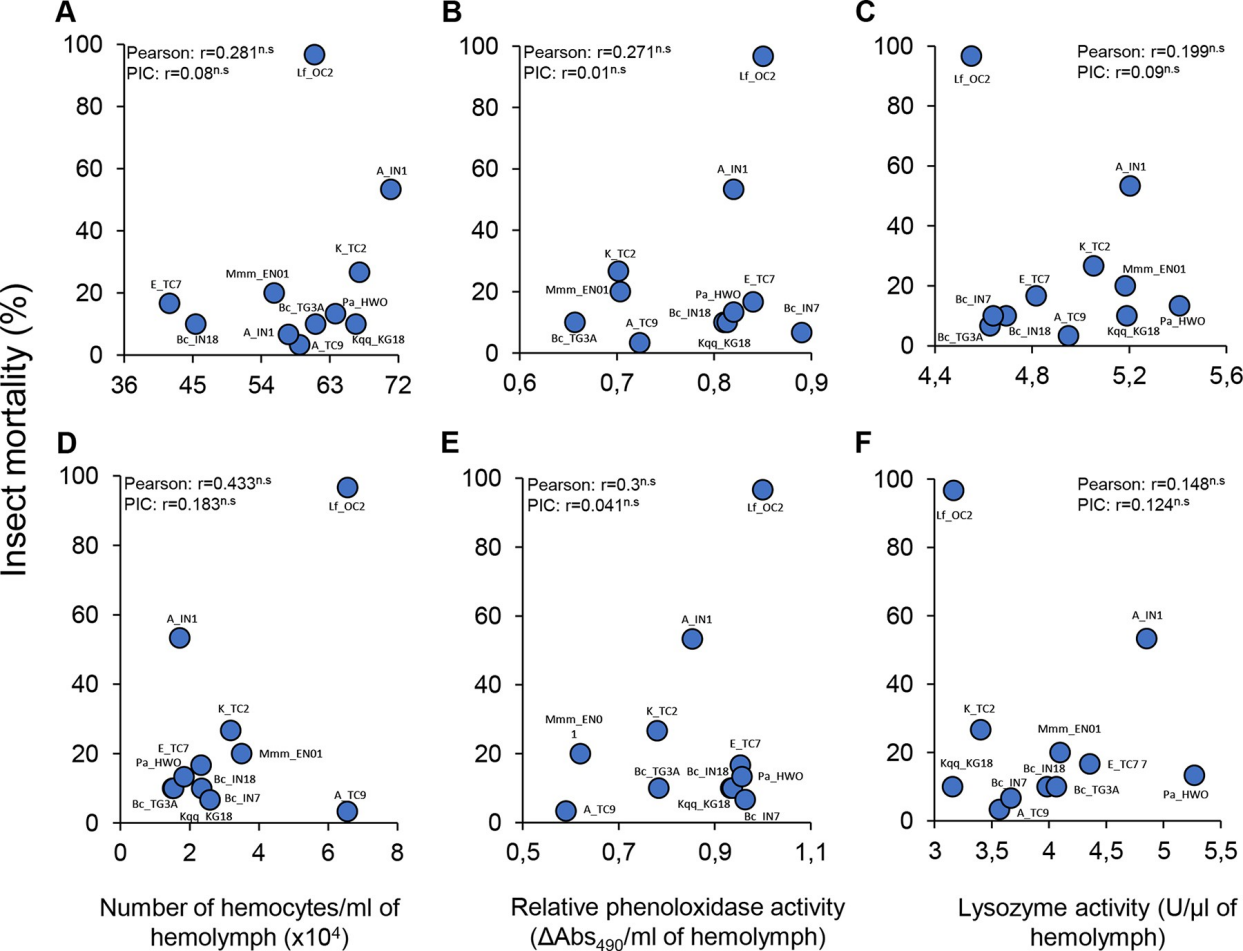
Relationship between insect immune responses to bacterial infection and bacteria pathogenesis. Correlation between insect mortality caused by bacterial infection 72h post-infection and: A) Mean hemocyte number per ml of hemolymph of *G*. *mellonella* larvae injected with different bacteria 4h post-infection; B) Mean lysozyme activity in the hemolymph of *G*. *mellonella* larvae injected with different bacteria 4h post-infection; C) Mean relative phenoloxidase activity per ml of hemolymph of *G*. *mellonella* larvae injected with different bacteria 4h post-treatment; D) Mean hemocyte number per ml of hemolymph of *G*. *mellonella* larvae injected with different bacteria 24h post-infection; E) Mean lysozyme activity in the hemolymph of *G*. *mellonella* larvae injected with different bacteria 24h post-infection; and F) Mean relative phenoloxidase activity per ml of hemolymph of *G*. *mellonella* larvae injected with different bacteria 24h post-treatment. Correlations were statistically assessed by person test and by independent phylogenetic contrast (PIC). n.s.: Statistically not significant.

No statistically significant correlations were observed between bacteria pathogenesis, measured as the insect mortality caused by bacterial infection 72h post-infection, and insect immune responses, measured as the number of hemocytes, lysozyme activity or phenoloxidase activity in the hemolymph of *G*. *mellonella* larvae injected with different bacteria 4h post-infection or 24h post-infection (Fig 5).

## Discussion

In this study, we isolated several bacterial strains from soil-borne nematodes and tested their pathogenesis against *G*. *mellonella* larvae and the immune responses of these insects to bacterial infection. The bacterial strains isolated were identified as *L*. *fusiformis*, *Kaistia* sp., *Alcaligenes* sp., *Enterobacter* sp., *K*. *quasipneumoniae* subsp. *quasipneumoniae*, *B*. *cereus*, *Acinetobacter* sp., *P*. *aeruginosa*, and *M*. *morganii* subsp. *morganii*.

Several of the bacterial species isolated in this study have been previously observed in association with soil-borne nematodes [73]. For instance, *Alcaligenes* sp., *Klebsiella* sp., *Acinetobacter* sp., *Pseudochrobactrum* sp., *Comamonas* sp., and *Leucobacter* sp. were isolated from specimens of *Rhabditis regina* [74]. Furthermore, numerous species of *Oscheius* are vectors of bacteria [75–77]. For instance, several species of *Acinetobacter*, *Bacillus*, *Klebsiella*, *Enterobacter*, *Pseudomonas*, *Lysinibacillus* and *Alcaligenes* have been isolated from different *Oscheius* species [30,78]. Recently, nematodes from two *O*. *tipulae* strains, TC2 and OC2, were found to efficiently kill *G*. *mellonella* larvae and *C*. *capitata* at different developmental stages [59]. The nematode-killing abilities could potentially be achieved by the pathogenic effects of the bacteria they carry.

In support of the above hypothesis, we observed that all the bacterial strains tested in this study, including some that were isolated from *O*. *tipulae* TC2 and OC2, were pathogenic and rapidly killed *G*. *mellonella* larvae. Among them, *L*. *fusiformis* Lf_OC2 isolated from *O*. *tipulae* (OC2) showed the highest effectiveness. *Lysinibacillus* species are ubiquitous [79]. In addition, *L*. *fusiformis* acts as a biocontrol agent against a wide range of plant diseases, due to its capacity to produce different antimicrobial compounds such as salicylic acid, chitinase, protease, and β-endoglucanase [80–83]. In contrast to our observations, other *L*. *fusiformis* strains have a low entomopathogenic effect against *Spodoptera exigua* Hübner (Lepidoptera: Noctuidae) [84]. However, other strains of this species are highly lethal to *Drosophila melanogaster* and show miticidal effect against *Varroa destructor* [85,86]. These previous literature reports together with our results indicate a great entomopathogenic potential of *L*. *fusiformis* against certain arthropods.

The entomopathogenic abilities of several other species isolated in this study have been documented in previous studies. This is the case of *P*. *aeruginosa* and *B*. *cereus*. *Pseudomonas aeruginosa* occupies diverse ecological niches [87]. It is an opportunistic pathogen of vertebrates, humans, insects and nematodes [88–93]. This bacterium is associated with nematodes such as *H*. *bacteriophora* and has been shown to kill other insects such as *Trichoplusia ni* [94]. On the other hand, *B*. *cereus* is widely distributed in many environments, and is used as a biocontrol agent against different plant diseases and insect pests [95–101].

The pathogenicity of several other bacteria isolated in this study has not been previously reported. This is the case of *K*. *quasipneumoniae* subsp. *quasipneumoniae* Kqq_KG18 isolated from *A*. *bodenheimeri* nematodes. This bacterial species is an ubiquitous opportunistic pathogen of humans and animals that is often found in soil, groundwater, animals, and plants [102]. However, closely related species such as *K*. *pneumoniae* and *K*. *oxytoca* kill *Trichoplusia ni* and *Culex pipiens*, respectively [94,103].

An important aspect of our study is regarding the type of association established between the bacterial strains isolated in this study and the nematodes. Soil-borne nematodes are frequently vectors of bacteria. *Oscheius* nematodes for instance carry different *Gammaproteobacteria* (*Enterobacter*, *Proteus*, *Providencia*, *Pseudomonas*, *Stenotrophomonas*), *Betaproteobacteria* (*Alcaligenes*), and *Bacilli* (*Bacillus*, *Enterococcus*, *Lysinibacillus*) [75,78]. Our experimental approach does not allow to determine whether the bacteria and the nematodes establish an obligate mutualistic symbiosis similar to that established by *Photorhabdus* and *Heterorhabditis* or by *Xenorhabdus* and *Steinernema* [10,11,104]. As several of the nematodes we studied here are soil-borne, bacteriophagus organisms, it is likely that they passible acquire the different bacteria while foraging and that the bacteria are carried on their cuticles or in their digestive tracts. Interestingly, we observed that some nematodes were associated with bacterial strains known to produce highly toxic metabolites, such as *M*. *morganii* subsp. *morganii* [105–109]. This observation opens up the question on whether these bacteria can also be detrimental to the nematodes. Our interpretation is that it is likely so, but it depends on bacterial densities. Under low bacterial densities, certain toxin-producing bacteria can associate with and be carried by the nematodes on their cuticle, and aid them killing soil-borne insects, while under high bacterial densities, nematodes might be killed by these bacteria. It is, therefore, possible that the nematodes have evolved mechanisms to avoid soil areas where toxic bacteria occur at high densities to avoid their toxic effects.

Insects strongly responded to the infection of all the bacteria tested in this study by increasing the number of hemocytes in the hemolymph, and also by activating key immune enzymes such as phenoloxidases and lysozymes. Interestingly, and contrary to our expectations, we observed no correlation between bacteria pathogenesis, measured as the insect mortality caused by bacterial infection, and insect immune responses, measured as the number of hemocytes, lysozyme activity, and phenoloxidase activity in the hemolymph of *G*. *mellonella* larvae injected with different bacterial strains. A potential explanation of these results is that insect immune responses might not be effective at suppressing the infection of certain bacterial species [110–113]. Additional experiments are required however to test this hypothesis and to understand the relative contribution of the different immune responses to limit bacterial infection.

## Conclusions

Bacteria associated with soil-borne nematodes have entomopathogenic capacities. From an applied perspective, our study motivates more research to determine their potential as biocontrol agents in environmentally friendly and sustainable agriculture.

## Supporting information

S1 Fig Phylogenetic tree based on ribosomal gene sequences of the bacteria isolated in this study and several related species. Phylogenetic relationships based on 16S rRNA gene sequences were inferred by using the Maximum Likelihood method based on the Kimura 2-parameter model. The tree with the highest log likelihood (-6121.67) is shown. The percentage of trees in which the associated taxa clustered together is shown next to the branches. The tree is drawn to scale, with branch lengths measured in the number of substitutions per site. There were a total of 932 positions in the final dataset. NCBI accession numbers of the sequences used for the analyses are shown.
(PDF)

S1 File (DOCX)
