# Supplementary material for: Entomopathogenic potential of bacteria associated with soil-borne nematodes and insect immune responses to their infection
Source: PLoS One. 2023 Jan 23;18(1):e0280675. doi: 10.1371/journal.pone.0280675 (PMC10045567; doi:10.1371/journal.pone.0280675)
Supplement: S1 Fig — Phylogenetic relationships based on 16S rRNA gene sequences were inferred by using the Maximum Likelihood method based on the Kimura 2-parameter model. The tree with the highest log likelihood (-6121.67) is shown. The percentage of trees in which the associated taxa clustered together is shown next to the branches. The tree is drawn to scale, with branch lengths measured in the number of substitutions per site. There were a total of 932 positions in the final dataset. NCBI accession numbers of the sequences used for the analyses are shown. (PDF) [file pone.0280675.s001.pdf]

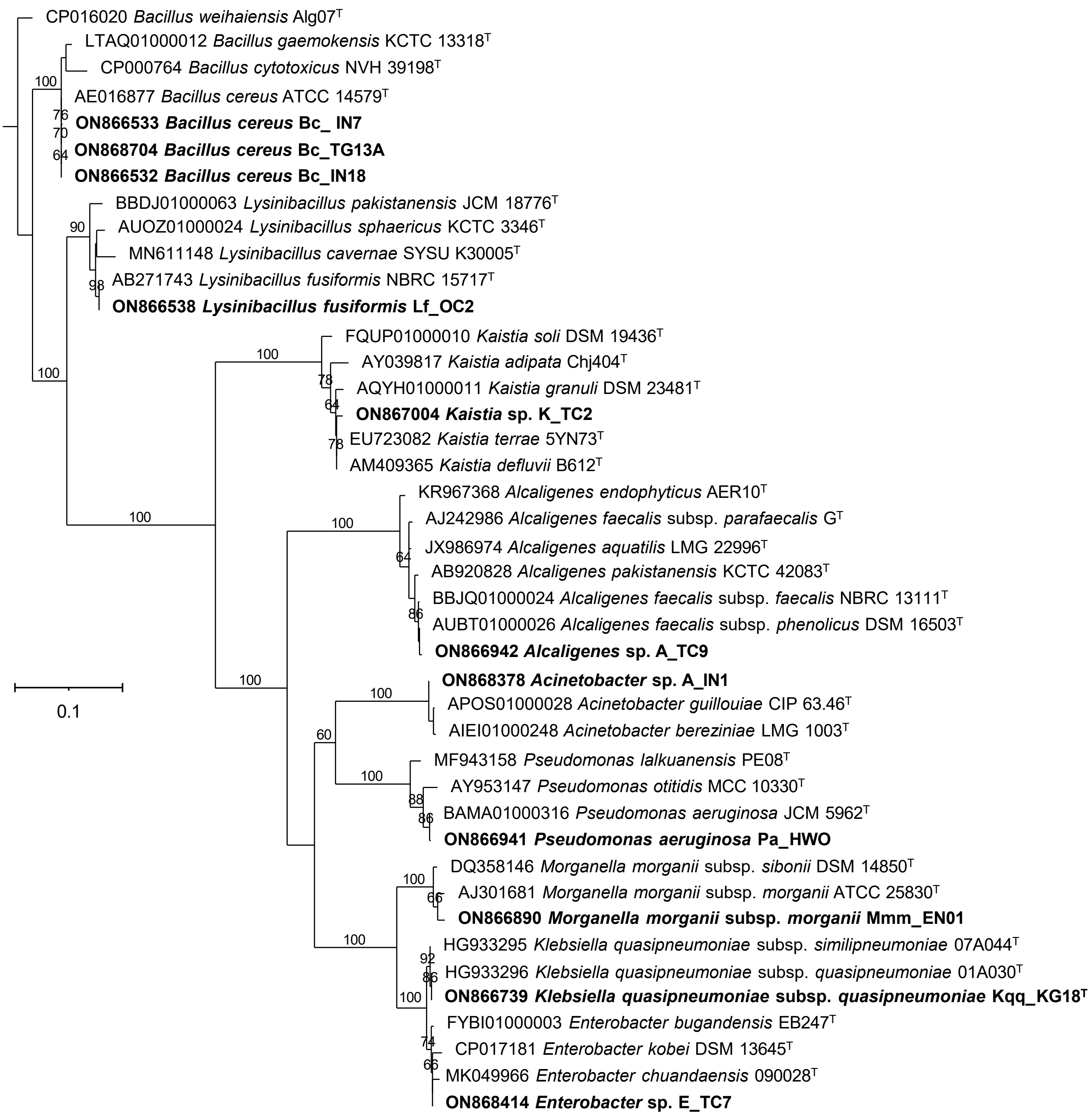

Figure S1. *Phylogenetic tree based on ribosomal gene sequences of the bacteria isolated in this study and several related species.* Phylogenetic relationships based on 16S rRNA gene sequences were inferred by using the Maximum Likelihood method based on the Kimura 2-parameter model. The tree with the highest log likelihood (-6121.67) is shown. The percentage of trees in which the associated taxa clustered together is shown next to the branches. The tree is drawn to scale, with branch lengths measured in the number of substitutions per site. There were a total of 932 positions in the final dataset. NCBI accession numbers of the sequences used for the analyses are shown.
