# Supplementary material for: Entomopathogenic potential of bacteria associated with soil-borne nematodes and insect immune responses to their infection
Source: PLoS One. 2023 Jan 23;18(1):e0280675. doi: 10.1371/journal.pone.0280675 (PMC10045567; doi:10.1371/journal.pone.0280675)
Supplement: S1 File — (DOCX) [file pone.0280675.s002.docx]

**Supporting information**

File. The data supporting the findings of this study can be found under this link: https://figshare.com/articles/dataset/data_article_bacteria_of_soil_borne_nematodes/21769868
